# Role of Polyphenols as Antioxidant Supplementation in Ischemic Stroke

**DOI:** 10.1155/2021/5471347

**Published:** 2021-06-25

**Authors:** Yuan Zhou, Shanshan Zhang, Xiang Fan

**Affiliations:** ^1^School of Basic Medical Sciences, Zhejiang Chinese Medical University, Hangzhou 310053, China; ^2^Key Laboratory of Neuropharmacology and Translational Medicine of Zhejiang Province, Zhejiang Chinese Medical University, Hangzhou 310053, China

## Abstract

Stroke is the second most common cause of death globally and the leading cause of death in China. The pathogenesis of cerebral ischemia injury is complex, and oxidative stress plays an important role in the fundamental pathologic progression of cerebral damage in ischemic stroke. Previous studies have preliminarily confirmed that oxidative stress should be a potential therapeutic target and antioxidant as a treatment strategy for ischemic stroke. Emerging experimental studies have demonstrated that polyphenols exert the antioxidant potential to play the neuroprotection role after ischemic stroke. This comprehensive review summarizes antioxidant effects of some polyphenols, which have the most inhibition effects on reactive oxygen species generation and oxidative stress after ischemic stroke.

## 1. Introduction

Stroke is the second most common cause of death globally and the leading cause of death in China [1, 2]. Approximately 71% of all stroke cases are ischemic stroke, and the proportion in the developed countries is estimated to be higher, reaching up to 85% [3]. Ischemic stroke is a group of acute cerebral vascular diseases caused by various reasons leading to the interruption of cerebral arterial blood flow and the corresponding brain tissue ischemia necrosis, resulting in the loss of neural function [4]. Ischemic stroke had a high incidence and mortality rate [5, 6], which seriously affects patient life quality and brings heavy mental and economic burden to the family of patients. Both diagnosis and treatment of stroke present enormous challenges. Stroke relevant biomarkers provide an important reference for the diagnosis and prognosis of stroke. An emerging study has found that irisin was an independent prognostic marker of ischemic stroke patients, whose decreased concentration is associated with poor outcome of patients [7]. Oxidative stress and inflammation related biomarkers from saliva of stroke patients also have drawn much attention of researchers [8].

Intravenous tissue plasminogen activator (tPA) thrombolytic therapy remains the only FDA-approved emergency drug treatment within 4.5 hours after acute ischemic stroke [9]. However, the increased risk of intracerebral haemorrhage and a short treatment time window limit tPA clinical application wildly [10, 11]. At present, defibrillating therapy, antiplatelet therapy, anticoagulant therapy, and neuroprotective therapy are all reported as the potential treatment of ischemic stroke. Still, all those therapies need more clinic evidence to confirm the efficacy. Therefore, it is of great significance to accelerate research and drug development on ischemic stroke to reduce the mortality rate and improve the life quality.

The pathogenesis of cerebral ischemia injury is complex; excitatory neurotoxicity, calcium overload, oxidative/nitrosative stress, and mitochondrial dysfunction are involved the main mechanisms of cerebral ischemia injury [12]. Cerebral ischemia induces cascade reactions with the overproduction of reactive oxygen species (ROS). Inherent antioxidant potential cannot neutralize ROS and keep the endogenous redox balance, which will cause oxidative stress. Oxidative stress plays an essential role in the fundamental pathologic progression of cerebral damage in ischemic stroke [13]. When oxidative stress occurs, ROS oxidizes lipids, proteins, and nucleic acids to damage cerebral tissue structure and cells [14, 15]. Oxidative stress can also cause neuronal apoptosis, inflammation, and blood brain barrier impairment, all of which will aggravate cerebral injury after ischemic stroke [16, 17]. Previous studies have preliminarily confirmed that oxidative stress should be a potential therapeutic target and antioxidant as a treatment strategy for ischemic stroke, even though the results of clinical trials are underwhelming [18, 19]. In recent years, polyphenols are the interesting natural products (from dietary vegetables, fruits, herb medicines, and so on) because of their beneficial effects on human health and diseases. Emerging experimental studies have demonstrated that polyphenols exert the antioxidant potential to play the neuroprotection role after ischemic stroke [20]. This comprehensive review summarizes antioxidant effects of some polyphenols, such as flavonoids, phenolic acids, curcuminoids, stilbenoids, and lignans, which have the most inhibition effects on ROS generation and oxidative stress after ischemic stroke.

## 2. Sources and Classes of Polyphenols

Just as its name implies, polyphenols are compounds characterized by more than one phenolic hydroxyl group. However, more views do not exclude compounds with only one phenolic hydroxyl group [21]. As secondary plant metabolites, polyphenols are mainly distributed in the bark, root, leaf, shell, and fruit of a plant. Thus, polyphenols are ubiquitous in daily necessities taking plants as raw materials, including vegetables, fruits, herb medicines, tea, red wine, part of food additives, and cosmetics.

**Figure 1:**
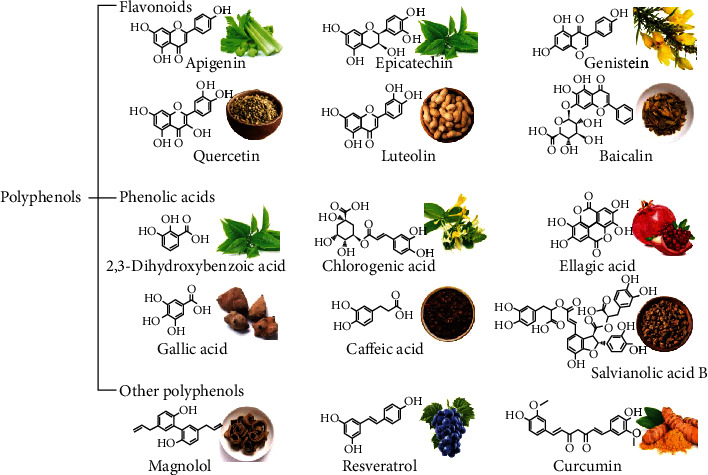
The chemical structures and plant origin of some common polyphenols and their classifications.

Among various natural products, flavonoids, phenolic acids, curcuminoids, stilbenoids, and lignans usually belong to polyphenols. However, not all natural products from these classifications meet the criteria for containing one or more phenolic hydroxyl groups. For instance, most of dibenzocyclooctene lignans from *Schisandra chinensis*, such as schisandrin, schisandrin B, schisandrin C, schisantherin A, schisandrol B, and gomisin G, have no hydroxyl group at benzene rings [22].  Figure 1 shows the chemical structures and plant origin of some common polyphenols and their classifications. It is worth mentioning that there are several subclasses as well. Flavonols, flavanones, flavones, flavan-3-ols, isoflavones, anthocyanins, dihydrochalcones, and proanthocyanidins all belong to the classification of flavonoids [23, 24]. Nevertheless, this article focuses on the antioxidant activity of polyphenols in ischemic stroke. Thus, Figure 1 does not distinguish compounds between these subclasses. In addition to ubiquitous in daily life, polyphenols are also known for their antioxidant property, which is also a core topic of discussion by nutritionists and medical workers [25].

## 3. Flavonoid

Flavonoids are among the most common natural products and widely present in various plants, including vegetables, fruits, and herbs. As dietary ingredients, cohort and case-control epidemiological studies confirmed that flavonoids could reduce the risk of cardiovascular disease and other chronic diseases [26]. More importantly, flavonoids are also primary active ingredients of many herbal medicines such as *Scutellaria baicalensis* and *Pueraria Lobata*, which showed significant pharmacological activities. Based on modern medicine studies, antioxidant activity is a relatively common effect of these flavonoids [27]. Thus, in traditional Chinese medicine prescription, antioxidant activity of flavonoids plays a more or less important role in various diseases. For instance, Angong Niuhuang Pill is widely used in the treatment of ischemic stroke clinically, and baicalin is proved to be a vital ingredient extracted from *Scutellaria baicalensis*, one of the 12 kinds of traditional Chinese medicines that make up Angong Niuhuang Pill [28]. This instance shows us the pharmaceutical value of flavonoids as antioxidant supplementation in ischemic stroke. Thus, in this section, antioxidant action and mechanism of each flavonoid monomer in treating ischemic stroke would be enumerated and discussed.

### 3.1. Baicalein and Baicalin

Baicalein is a flavone with three hydroxy groups at positions C-5, C-6, and C-7. Baicalin is the 7-O-glucuronide of baicalein. The same as baicalin mentioned in a previous paragraph, baicalein is also an essential active ingredient from *Scutellaria baicalensis* [29]. Directly, baicalein could protect middle cerebral artery occlusion (MCAO) rats from ischemia/reperfusion (I/R) injury to some degree [30, 31]. Neurological severity, infarct volume, brain water content, and Evans blue leakage level proved the therapeutic effects of baicalein on ischemic stroke as well [30, 31]. Additionally, many common indicators related to oxidative stress suggested that antioxidant action of baicalein played a significant role. Reactive oxygen species (ROS), malondialdehyde (MDA), and 8-hydroxy-2′-deoxyguanosine (8-OhdG) were reduced, while the levels of NADPH, quinone oxidoreductase-1 (NQO1), glutathione peroxidase (GSH-Px), superoxide dismutase (SOD), glutathione (GSH), and catalase (CAT) were significantly increased after baicalein treatment [30]. The activation of adenosine 5′-monophosphate- (AMP-) activated protein kinase (AMPK) and nuclear factor E2-related factor 2 (Nrf2) signaling pathways is the crucial mechanism for this antioxidant effect [30]. And upregulation of mitochondrial membrane potential (MMP) might be another mechanism that baicalein prevents neuronal cells from oxidative stress injury [31]. Mouse hippocampal neuronal cell line HT22 and toxic material iodoacetic acid were applied to establish oxidative injury cell model, whose cell survival rate could be improved by baicalein [32].

Baicalin exhibited a similar antioxidant effect as baicalein to ischemic stroke, which could also be speculated by their identical chemical structure skeleton. In baicalin research, mitochondrial succinate dehydrogenase (SDH) was a novel enzyme, which could stimulate excessive ROS production and abnormal glutamine synthetase degradation in astrocytes of ischemic stroke rats [33]. Baicalin was able to prevent astrocytes from oxidative stress via inhibiting SDH [33]. Additionally, baicalin could downregulate the levels of superoxide and peroxynitrite [34].

### 3.2. EC, ECG, and EGCG

(-)-Epicatechin (EC), (-)-epicatechin gallate (ECG), and (-)-epigallocatechin gallate (EGCG) are three representative polyphenols from green tea. As shown in Figure 1, the chemical structure of EC is the most simple, ECG is an ester derived from a (-)-epicatechin and a gallic acid. Very similar to ECG, EGCG is another ester with one more hydroxyl group. These tea polyphenols exert ubiquitous antioxidant effects as tea is a standard beverage in our daily life. Researchers also have explored their potential in treating ischemic stroke and indicated that EGCG was a promising antioxidant supplement or ancillary drug in stroke treatment. Several pieces of literature reported antioxidative and other roles of EC and ECG as well.

EGCG exhibited significant protective effects on various MCAO animal models. Neurologic severity score, infarct volume, and expression of apoptosis-related proteins collectively proved this effect [35–38]. ROS, MDA, SOD, CAT, and other oxidative stress-related indicators all revealed antioxidant activity of EGCG [35–38]. The activation of Nrf2-antioxidant responsive element (ARE) signaling pathway might be the main mechanism of antioxidant activity of EGCG. Nrf2-ARE is a classical antioxidant signaling pathway, and many natural products target it to treat nervous system diseases [22]. EGCG was reported to upregulate Nrf2 expression, thereby mitigating oxidative stress damage and promoting angiogenesis [35, 36]. The function and mechanism of EC in treating ischemic stroke were both analogous to EGCG, and the detailed information can be found in Table 1. ECG could protect human brain microvascular endothelial cells (HBMECs) from oxygen-glucose deprivation/reoxygenation- (OGD/R-) induced injury, while inhibition of autophagy and promotion of angiogenesis contributed to this protective effect [39]. Antioxidation also supported this protective effect, and the reduction of ROS provided direct evidence [39].

### 3.3. Quercetin

Quercetin is another well-known and widespread flavonol with antioxidant activity. More than 100 kinds of herbs, such as *Flos Sophorae Immaturus*, *Cacumen biotae*, and *Alpinia officinarum Hance*, are rich in quercetin. Vegetables such as onion, fruits such as apple, and drinks such as red wine were all detected to have a certain amount of quercetin [40]. Therefore, quercetin is a noteworthy antioxidant supplement in our daily life.

Quercetin protected against cerebral ischemia/reperfusion injury, and antioxidant activity plays a vital role [41]. Besides, quercetin suppressed lipopolysaccharide- (LPS-) induced adhesion molecule expression to treat atherosclerosis, which is a crucial induction factor of ischemic stroke. Researchers found that quercetin was able to activate Nrf2, thereby upregulating heme oxygenase 1 (HO-1) expression. Intriguingly, though adhesion molecule was under the regulation of HO-1, this phenomenon was antioxidant-independent. Nevertheless, Nrf2 activation and HO-1 upregulation actually increased antioxidants and played a role in reducing cell damage [42].

### 3.4. Astragaloside IV

Astragaloside IV is a principal component of a common herb clinically used for ischemic stroke: *Radix Astragali*. The pharmacological effect and mechanism studies of astragaloside IV to ischemic stroke are numerous and diverse [43–46]. Neurocyte protection, blood brain barrier protection, intestinal microbiota regulation, and mitochondrial function recovery were all referred to in relevant literature [43–46]. Meanwhile, antioxidant activity of astragaloside IV was correlation to all these effects.

### 3.5. Genistein

Genistein is a plant estrogen wildly distributed in many legumes. As a potential compound in treating ischemic stroke, genistein plays a role in neuroprotection. Researchers illuminated that genistein was able to activate Nrf2 to upregulate several antioxidase expressions [47]. So the ROS level could be decreased by genistein, and its stimulation to nuclear factor kappa-B (NF-*κ*B), c-Jun N-terminal kinase (JNK), and extracellular regulated protein kinases (ERK) signaling pathways could be alleviated [48, 49]. As a result, nerve injury caused by inflammation and apoptosis would be partially mitigated. Stroke-prone spontaneously hypertensive rat models also suggested the antioxidant activity of genistein. Aortic endothelial cells from these rats have been detected to have a lower level of nicotinamide adenine dinucleotide phosphate (NADPH) after genistein treatment. And the authors declared that downregulation of p22phox and angiotensin II type 1 receptor expression resulted from genistein played an antioxidative role at the transcription level [50].

### 3.6. Other Flavonoids

Besides the flavonoids enumerated above, many flavonoids were reported to exert antioxidant function and show definite potential as supplementations in ischemic stroke (see Table 1).

Rutin is a flavonoid glycoside formed by quercetin and a disaccharide. In the differentiation process of human neuroblastoma cells (IMR32) induced by retinoic acid, rutin could decrease ROS level [51].

Hesperidin is a flavonoid found in citrus fruits, which could observably improve the content of antioxidase, such as CAT, SOD, and GSH, in rats or mouse ischemic stroke models. Cerebral injury and abnormal behavior were mitigated as well [52, 53]. Neohesperidin has the same molecular weight and very similar chemical structure as hesperidin and is also abundant in citrus fruits. Neohesperidin activated the Akt/Nrf2/HO-1 signaling pathway to inhibit oxidative stress and protect MCAO-induced brain damage [54]. Another flavonoid from citrus fruits, tangeretin, with five methoxy groups and no oxhydryl on its flavone skeleton, prevented HBMECs from OGD-induced injury via inhibiting the JNK signaling pathway. And oxidative stress injury was attenuated as well [55]. As a matter of fact, the crosstalk between JNK and Nrf2 signaling pathways plays a role in oxidative damage of stroke [56]. Naringenin, abundant in citrus fruits, exerted antiapoptotic and antioxidant effects in both the OGD/R cell model and MCAO rat model. Nrf2 gene silence or overexpression impacted these two effects, which proved the pivotal role of Nrf2 for naringenin treating ischemic stroke [57]. Another flavonoid from citrus fruits, nobiletin, showed potential in treating ischemic stroke for its anti-inflammatory and antioxidant activities [58].

Apigenin is a trihydroxyflavone widely distributed in celery and other vegetables. As a common compound with antioxidant activity, apigenin was also adopted to treat ischemic stroke as an attempt. The antioxidant function of apigenin contributed to its neuroprotective effect, and increased MMP might be a notable phenomenon [59, 60]. An apigenin flavone glycoside, vitexin, exerts stroke-treating effect primarily through reversing autophagy dysfunction. In the process of experiment, oxidative damage indexes also suggested the antioxidation of vitexin [61].

Isoquercetin is derived from quercetin with a *β*-D-glucosyl residue attached at position 3. Isoquercetin activated Nrf2 to exert an antioxidant effect, while toll-like receptor 4 (TLR4), NF-*κ*B, and mitogen-activated protein kinase (MAPK) signaling pathways participated in neuroprotection as well [62]. It is worth mentioning that Nrf2 gene transcription and protein expression were both upregulated [63]. Isorhamnetin played a role in diabetic stroke. In methylglyoxal plus OGD-induced cell model (HBMECs), isorhamnetin exhibited antioxidative, anti-inflammatory, and antiapoptotic effects [64].

Phloretin is a dihydrochalcone that belongs to flavonoid, which could protect rats from ischemia/reperfusion (I/R) injury via Nrf2 activation [65]. A phytoestrogen biochanin A shows a similar effect as phloretin [66]. Morin, a pentahydroxyflavone commonly used as a natural dyestuff, could reduce ROS, inhibit lipid peroxidation (LPO), and protect blood brain barrier integrity [67]. Breviscapine, also termed scutellarin, was widely used in the clinic to exert anticoagulation and vasodilation effects. In the rat MCAO model, breviscapine could improve cognitive competence and protect the nervous system. Antioxidant and anti-inflammatory effects of breviscapine played a pivotal role [68].

Hispidulin is analogous to baicalin in chemical structure, with a methoxy group at position 6 instead of oxhydryl. Hispidulin activated Nrf2 in I/R rats through regulation of AMPK/glycogen synthase kinase-3*β* (GSK3*β*) signaling. Nrf2 gene knockdown decreased the neuroprotective effect of hispidulin, and AMPK inhibitor downregulated expression of Nrf2 [69]. Myricetin is a flavone extracted from the leaves of *Myrica rubra*, which could treat ischemic stroke via improving antioxidase expression and mitochondrial function. Mitochondrial ATP and MMP increased while ROS and MDA in mitochondria decreased after myricetin treatment. And researchers discovered that activating Nrf2 was the critical mechanism [70]. Xanthoangelol is an important ingredient of propolis, which triggered Nrf2 to treat ischemic stroke [71].

Kaempferol protected rats from I/R injury, p-Akt, p-GSK3*β*, Nrf2, p-NF-*κ*B, and oxidative stress, and inflammation-related proteins were detected, and the levels of those protein expressions were regulated by kaempferol [72]. Two flavonoids extracted from herb *Herba epimedium*, icariin and icariside II, were able to scavenge ROS, respectively. Icariin activated the Nrf2/sirtuin-3 (SIRT-3) signaling pathway, while icariside II inhibited NADPH oxidase activity [73, 74]. A quercetin and an *α*-L-rhamnosyl moiety formed quercitrin, which reduced ischemic stroke injury via inhibiting platelet activation in arterial thrombosis. Antioxidation of quercitrin was observed as well, and inhibition of TNF receptor-associated factor 4 (TNAF4)/p47^phox^/Hic5 axis was the reason [75].

Sanggenon C could protect rats from MCAO injury through inhibition of ras homolog gene family. The member A/rho-associated protein kinase (RhoA/ROCK) signaling pathway benefited the anti-inflammatory and antioxidant properties of sanggenon C. The reduced efficacy of sanggenon C after RhoA overexpression illustrated this [76]. Additionally, xanthohumol, luteoloside, pinocembrin, scutellarin, silibinin, chrysin, and other flavonoids were all reported to exhibited antioxidant activity in treating ischemic stroke [21].

## 4. Phenolic Acid

Phenolic acids are secondary metabolic products of plants and therefore widely exist in herbs, vegetables, and fruits. Structurally, phenolic acids have one benzene ring and one or more than one phenolic hydroxyl group. In comparison to flavonoids, study progress about phenolic acids was relatively later and slower. Nevertheless, some phenolic acids, such as salvianolic acid B (from famous herb medicine *Salvia miltiorrhiza*) and chlorogenic acid (from *Lonicera japonica*), aroused great interests of researchers [95–97]. In general, phenolic acids have shown potential in antioxidation, antitumour, and antibiosis. We have highlighted the correlation between oxidative stress injury and ischemic stroke. Thus, as we expected, some phenolic acids played an antioxidative role in treating ischemic stroke.

### 4.1. Ferulic Acid

Natural ferulic acid often binds to polysaccharides or proteins to form the skeleton of plant cell wall [98]. Thus, many plants, such as *Ferula asafoetida*, *Angelica sinensis*, and onion, contain abundant ferulic acid. As a proverbial free-radical scavenger, ferulic acid was widely used in the food industry (antioxidant) and cosmetic industry (antiageing) [99]. In ischemic stroke, ferulic acid could decrease the content of LPO product 4-hydroxynonenal (4-HNE) and DNA oxidative damage marker 8-OhdG [100]. The researchers declared that reducing the expression of intercellular cell adhesion molecule-1 (ICAM-1) mRNA mitigated oxidative damage. With ICAM-1 inhibition, the number of microglia/macrophages decreased and thereby showed considerable anti-inflammatory action. Ultimately, inflammation-induced oxidative stress and apoptosis were ameliorated [100]. Another literature reported peroxiredoxin-2 and thioredoxin, as antioxidant protein, have a strong neuroprotective effect. Ferulic acid significantly improved the protein expression of peroxiredoxin-2 and thioredoxin detected by proteomics in the brain of MCAO rats [101].

### 4.2. Gallic Acid

Gallic acid is another phenolic acid applied in a vast range of foods and cosmetics. Gallic acid is a trihydroxybenzoic acid with three hydroxy groups located at positions 3, 4, and 5. In Na_2_S_2_O_4_-induced hypoxia/reoxygenation SH-SY5Y cells, MMP, mitochondrial ROS, ATP level, oxygen consumption, and mitochondrial permeability transition pore viability all suggested that gallic acid exhibited a powerful effect in restoring mitochondrial dysfunction. And this effect was naturally beneficial to maintain cellular redox balance [102]. Another literature reported the function of gallic acid in treating post-stroke depression. The strong correlation between behavioral parameters and antioxidant enzyme levels such as SOD and GSH before and after gallic acid treatment proved the critical role of antioxidation. Additionally, a gallic acid derivative, methyl-3-O-methyl gallate, showed better antioxidant and antidepressant effects than gallic acid [103]. This result reminded us gallic acid might be a lead compound in the pursuit of a more efficient antioxidant to treat ischemic stroke. High concentrations of particulate matter might increase risk of ischemic stroke. To dusty particulate matter exposed stroke rats, gallic acid also exerted observably antioxidant effect [104].

### 4.3. Salvianolic Acid B

In China, Composite Salvia Miltiorrhiza Injection is a marketed drug approved by National Medical Products Administration for cerebrovascular accident prevention and treatment. Salvianolic acid B is one of the most important ingredients of Composite Salvia Miltiorrhiza Injection [105]. A metabolomics study suggested salvianolic acid B has antioxidant function because of content changes of oxidative stress-related biomarkers [95]. Researchers also discovered that salvianolic acid B could suppress activation of astrocytes and microglia and downregulate the ROS level in MCAO mice [96].

### 4.4. Other Phenolic Acids

Chlorogenic acid is a depside formed by caffeic acid and quinic acid. Literatures about antibacterial, antiviral, and antioxidant effects of chlorogenic acid are numerous [106]. Researchers also made an effort to study the function of chlorogenic acid in ischemic stroke. One literature reported that chlorogenic acid dose-dependently improved learning and memory ability and alleviated brain damage of I/R rats. Proteins in the Nrf2 signaling pathway, including Nrf2, HO-1, and NQO1, were all detected at a higher level after administering chlorogenic acid. The authors also used Nrf2 inhibitor ML385 to further prove the effect of chlorogenic acid in activating the Nrf2 signaling pathway [97].

Caffeic acid could cross blood brain barrier and has multiple biological activities including antioxidation [107]. Caffeic acid could ameliorate neurological dysfunction and decrease infarct volume after focal cerebral ischemia in rats by downregulating expression of 5-lipoxygenase, an enzyme catalyzing lipid oxidation [108, 109]. In the process of arachidonic acid producing leukotrienes, 5-lipoxygenase exerted key catalysis, which exacerbated nerve damage of cerebral ischemia rats [108].

Compared to other phenolic acids, ellagic acid has a characteristic organic heterotetracyclic structure. Antioxidant and antiproliferative effects of ellagic acid are the most concerned. As an active ingredient in cranberries, strawberries, pomegranates, and other common fruits, the role of ellagic acid in ischemic stroke is also worth investigating [110]. As expected, ellagic acid showed a beneficial effect on MCAO rats through regulating the expression of zonula occludens-1 (up), aquaporin 4, and matrix metalloproteinase 9 (down). The high level of some representative antioxidant enzymes also clarified antioxidation of ellagic acid [111].

Rosmarinic acid could also upregulate Nrf2 to exert antioxidant and neuroprotective functions. Zinc protoporphyrin IX, an HO-1 inhibitor, suppressed the antioxidant and antiapoptotic effects of rosmarinic acid. And researchers discovered that phosphoinositide 3-kinase (PI3K)/Akt was an upstream regulator of Nrf2 and PI3K inhibitor LY294002 decreased Nrf2 and HO-1 expression. To sum up, rosmarinic acid protected against ischemic stroke; PI3K/Akt signaling pathway activation and following up-regulation of Nrf2 and HO-1 were the involved mechanism [112].

## 5. Curcuminoid

Curcumin, demethoxycurcumin, tetrahydrocurcumin, hexahydrocurcumin, and bisdemethoxycurcumin belong to curcuminoid family. Curcumin was extracted from *Curcuma longa* at the earliest. Curcumin had extensive and strong pharmacological effects, such as anti-inflammation, antioxidation, and antitumour [113, 114]. In recent years, more and more evidence showed that curcumin had pretty high potential in treating cardiovascular and cerebrovascular diseases [115]. And kind of literature reported the effects of curcumin against ischemic stroke.

Thiyagarajan and Sharma firstly reported that the antioxidation-mediated neuroprotective effect was why curcumin protected rats from I/R injury in 2004. Concretely, dose-dependent reduction of cerebral infarct volume and cerebral edema volume proved the effect of curcumin against ischemic stroke. Peroxynitrite formation inhibition and protein tyrosine nitration reduction in the cytosolic suggested the antioxidative role of curcumin [116]. Another literature in 2010 made a similar study and mainly focused on Caspase 3, B-cell lymphoma-2 (Bcl-2), and other apoptosis-related proteins. MDA was downregulated as an oxidative stress index as well [117].

Molecular mechanism of antioxidant activity of curcumin in ischemic stroke was complicated. AMPK/uncoupling protein 2 (UCP2) signaling pathway was referred to as UCP2 which was able to limit excess ROS. Researchers found that curcumin could upregulate p-AMPK and UCP2, by which cerebrovascular and endothelial cell dysfunction could be attenuated. AMPK inhibitor, UCP2 inhibitor, and UCP2 gene knockout all suggested significance of this pathway to antioxidation of curcumin [118, 119]. The effect of curcumin on AMPK was also related to endoplasmic reticulum stress and associated thioredoxin-interacting protein/NACHT, LRR, and PYD domain-containing protein 3 (TXNIP/NLRP3) inflammasome activation. And endoplasmic reticulum stress in this paper resulted from a high ROS level [120]. Akt/Nrf2 was also involved in the antioxidant property of curcumin. Impacting on Akt phosphorylation was regarded as the critical factor of antioxidant and neuroprotective effects of curcumin [121]. Another report highlighted the role of peroxiredoxin 6 and specific protein1 (SP1) after ischemic stroke. The peroxiredoxin 6 gene silence or SP1 antagonism would severely weaken the therapeutical effect of curcumin. In normal conditions, the number of peroxiredoxin 6-positive neuronal cells and protein expression of peroxiredoxin 6 were both increased after curcumin administration [122]. Additionally, enhancement of apurinic/apyrimidinic endonuclease 1 in level and activity by curcumin also benefited its oxidation resistance and therapeutic effect [123].

Tetrahydrocurcumin is a derivant of curcumin, with double bonds be reduced to single bonds. Researchers found that tetrahydrocurcumin also plays a role in ischemic stroke, and recovering mitochondrial dysfunction of cerebral vascular cells might be a key factor [124, 125]. Other than common indexes such as neurological score, brain edema, cerebral infarction, and blood flow, the authors also found that tetrahydrocurcumin reduced permeability of blood brain barrier and recovered abnormal homocysteine metabolism via altering several related enzymes [124]. More importantly, tetrahydrocurcumin alleviated mitochondrial oxidative stress and inhibited mitochondrial dysfunction induced by oxidative stress. Levels of thioredoxin-2, SOD2, p47^phox^, and gp91^phox^ all proved the effect of tetrahydrocurcumin [124].

Hexahydrocurcumin, as one of the major metabolites of curcumin, significantly reduced the neurological deficit scores and the infarct volume in cerebral I/R injury rats. Treatment with hexahydrocurcumin significantly attenuated oxidative stress and inflammation, with decreased levels of MDA and NO and increased levels of the antioxidative enzymes and superoxide dismutase (SOD) activity in I/R rats [126]. A comparative study demonstrated that pretreated with polymeric *N*-isopropylacrylamide (PNIPAM) nanoformulation of curcumin, demethoxycurcumin, and bisdemethoxycurcumin intranasal delivery significantly improved neurological deficits, locomotor activity, and grip strength by decreasing the level of LPO and increasing the activities of antioxidant enzymes (GSH-Px, glutathione reductase, SOD, and CAT) in MCAO rats. These results suggest that PNIPAM-loaded curcumin nanoparticles may also be a potential neuroprotective agent against various conditions where cellular damage is a consequence of oxidative stress [127].

## 6. Stilbenoid

Resveratrol is a classic biologically active natural product, also a well-known and widely used antioxidant. Many literatures described stilbenoid as a nonflavonoid polyphenol. And more precisely, it was classified as a stilbenoid, which took stilbene or its polymeride as a skeleton structure. Resveratrol has both *cis* and *trans* structures, and *trans* structures are the abundant existing form in plants.

For patients who had a stroke within one year, oral 100-200 mg resveratrol daily for one year reduced or limited rising of blood pressure, blood glucose, blood lipid, and body mass index, which suggested resveratrol positively affected the prognosis of stroke patients [128]. As an antioxidant, resveratrol was applied to treat ischemic stroke in an experimental study as well. Sinha et al. firstly indicated that resveratrol protected MCAO rats via inhibition of oxidative stress [129]. Numerous relevant cell models and animal models were then adopted and proved the antioxidative role of resveratrol during ischemic stroke. H_2_O_2_-induced oxidative stress injury in hippocampal slice was alleviated by resveratrol, with an improved level of GST [130]. Similarly, in OGD-injured PC12 cells, bilateral common carotid artery (BCCA) occlusion induced cerebral infarction rats and diabetic rats with ischemic stroke; resveratrol exhibited antioxidant activity as well [131–133]. Enriched environment is beneficial to the recovery of brain injury and neurological dysfunction after ischemic stroke. In enriched environment, the therapeutic effect of resveratrol was improved, and the antioxidation could still be observed [134]. Hermann et al. divided the administration modes of resveratrol into prophylactic delivery, acute delivery, and postacute delivery, corresponding to administrating daily for 7 days until surgery, administrating immediately after reperfusion and 24 h after reperfusion, and administrating daily for 28 days after surgery, respectively. The result suggested that the first two administration modes were conducive to exert an antioxidant effect, while in administration after surgery group, thiobarbituric acid reactive substances (TBARS) formation and HO-1 level showed no significant difference with the control group [135].

The antioxidation mechanism for resveratrol mainly includes restoring mitochondrial function, activating sirtuin-1 (SIRT-1) and Nrf2. The significance of mitochondria to maintain redox homeostasis was mentioned above. In the oxygen and nutrient-deficient environments, excessive ROS production by the mitochondria would lead to mitochondrial lipid peroxidation and mitochondrial membrane depolarization. Thus, a vicious cycle of mitochondrial damage and oxidative stress would ultimately aggravate ischemic stroke [136]. Genomic analysis was applied in the neuronal-astrocytic coculture model to detect the gene expression differences before and after resveratrol preconditioning. TCA cycle, oxidative phosphorylation, and pyruvate uptake-related genes were upregulated. ATP level, glycolysis, and mitochondrial respiration efficiency were observed to be increased by resveratrol as well. All this evidence collectively illuminated the protection of resveratrol from oxygen and nutrient-deficient induced mitochondrial dysfunction [137, 138].

SIRT-1 is a deacetylase that regulated various biological functions of substrate proteins by deacetylation. Cell metabolism and apoptosis were involved in the regulating effect of SIRT-1 [139]. Concretely and correlatively, mitochondrial biosynthesis and fatty acid oxidation regulated by SIRT-1 were concerned in this paper [140]. Resveratrol activated SIRT-1, thereby protecting endothelium of the cerebrovasculature from oxidative stress injury, and ultimately exhibited a curative effect on ischemic stroke. SIRT-1 inhibitor significantly blocked these functions of resveratrol [141]. In terms of mechanism, activated SIRT-1 could increase the expression of downstream protein peroxisome proliferator-activated receptor *γ* coactivator-1*α* (PGC1*α*) and target genes UCP2 and SOD2 [142]. Resveratrol could also activate Nrf2 and upregulate the expression of downstream proteins, including HO-1 and NQO1, which was verified by multiple research groups based on the OGD cell model and MCAO animal model [143–145].

## 7. Other Polyphenols

Lignan is a kind of phytoestrogen formed by two phenylpropanoid derivatives [22]. Magnolol belongs to the lignan family and exhibits antioxidant property during ischemic stroke. As shown in Figure 1, magnolol is a typical polyphenol and abundant in herb medicine *Magnolia officinalis*. Magnolol showed a certain degree of competence in scavenging free radicals. As for lipid peroxidation inhibition in brain tissue, magnolol exhibited strong ability, and the IC50 value of dPPH radical scavenging assay was lower than *β*-estradiol, *α*-tocopherol, and ascorbic acid. MDA and 4-HNE level, nitrate/nitrite, and myeloperoxidase (MPO) activity all verified this. A direct neuroprotective effect was reflected in neonatal rat hippocampal slice cultures, and magnolol could mitigate the damage induced by OGD [146].

## 8. Conclusion and Prospect

### 8.1. Molecular Mechanisms of Polyphenols as Antioxidant Supplementations

Based on the introduction and Table 1, up to now, reported works observed antioxidation of polyphenols via detection of ROS, antioxidation-related enzymes such as SOD, CAT, GSH, and GSH-Px and oxidative stress byproducts such as MDA, 4-HNE, and 8-OhdG. As for molecular mechanisms, Nrf2-ARE was a recognized signaling pathway under regulation of polyphenols, including flavonoids, phenolic acids, curcuminoids, and stilbenoids. Specifically, curcumin-activated AMPK/UCP2 signaling pathway and resveratrol-activated SIRT-1 are both beneficial to antioxidation. The regulation effects on these three pathways were all repetitively verified by specific inhibitors or gene knockout.

**Figure 2:**
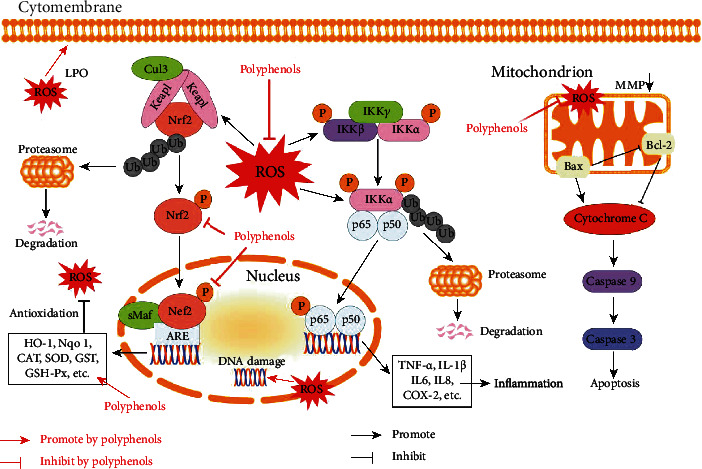
Molecular mechanisms of polyphenols as antioxidant supplementations in ischemic stroke.

Figure 2 exhibits the correlation of ROS and Nrf2-ARE signaling pathway. Under condition of redox equilibrium, Nrf2 usually locates in the cytoplasm and is limited by an upstream regulator Keap1, an E3 ubiquitin ligase complex. Keap1 catalyzes ubiquitin modification of Nrf2 cooperatively with Cullin-3 (Cul3) and subsequently degraded by the 26 s proteasome. Under oxidative stress, high ROS-induced electrophile metabolites would modify nonenzymatic covalent highly reactive cysteine residues in Keap1. Ultimately, Nrf2 would be released by Keap1 and exert its biological function. As a transcription factor, Nrf2 transfers to the nucleus and forms a dipolymer with small musculoaponeurotic fibrosarcoma (sMaf), which would bind to ARE and promotes expression of a series of antioxidation-related enzymes such as HO-1 and Nqo1. And cytoplasm Nrf2 plays a role via phosphorylation, nuclear localization, and ARE binding [147–149]. In the literatures about polyphenols treating ischemic stroke, Nrf2 transcriptional, expression, and phosphorylation level were all reported to be upregulated, which illuminated the role of Nrf2 in the antioxidation of polyphenols.

The high level of ROS not only stimulates Keap-Nrf2 but also activates inflammation and apoptosis-related signaling pathways.  Figure 2 also shows the crosstalk between ROS and NF-*κ*B, a significant inflammatory regulator, which is held in a resting state through association with inhibitor of *κ*B (I*κ*B) proteins [150]. On the one hand, some enzymes, such as gp91 phox, inducible nitric oxide synthase (iNOS), and cyclooxygenase-2 (COX-2) that regulated by NF-*κ*B, also play a role in promoting ROS. On the other hand, ROS could trigger the activation of the I*κ*B kinase complex, leading to phosphorylation, ubiquitination, and degradation of I*κ*B proteins, which could release anti-inflammatory factors and induce inflammation [151, 152]. Actually, in many experiments, NF-*κ*B was observed to be activated, and various inflammatory factors such as interleukin-1*β* (IL-1*β*), interleukin-6 (IL6), interleukin-8 (IL8), and tumour necrosis factor *α* (TNF-*α*) were increased [152]. Thus, downregulation ROS effect of polyphenols was an important reason for inflammation mitigation in stroke patients.

Elevated ROS in mitochondria would initiate the apoptotic process. Concretely, as shown in Figure 2, the high level of BCL2-associated X (Bax) and the low level of Bcl-2 along with loss of MMP, leading to apoptosome formation and thereby downstream caspase cascade, ultimately induced apoptosis [153]. And the level changes of Bax, Bcl-2, MMP, and Caspase 3 were all detected in stroke cell or animal models after polyphenols administration.

### 8.2. Superiorities of Polyphenols as Antioxidant Supplementations

Until now, there is no efficacious drug for ischemic stroke treatment besides tPA thrombolytic therapy. Many potential compounds were abandoned after phase II or III clinical trials because of inadequate efficacy, overstrong side effects, or other reasons. Among these failed compounds, inhibition of free radical production, free radical scavenging, free radical degradation, and mitochondrial targeted antioxidation were all taken as antioxidant strategies [154]. The temporary failure of antioxidants was disappointing. Nevertheless, stroke mortality showed a downward trend, and effective prevention and rehabilitation might be a significant reason [155]. During prevention and rehabilitation of ischemic stroke, healthy diet and exercise played a nonnegligible role. As described above, as a class of natural products, polyphenols were abundant in fruits, vegetables, and drinks, which exhibited the critical advantage of polyphenols: convenient ingestion and subtle influence.

Not only exerting antioxidative function in daily life diet, some polyphenols were also applied in clinical settings for a long time. Taking baicalin for instance, its source herb *Scutellaria baicalensis* has been used as ischemic stroke medication in China. Other polyphenols from herb medicine might likewise play a role in ischemic stroke. Inherited clinical experience ensured the efficacy and safety of these polyphenols to a certain degree, which was another advantage of polyphenols. Nevertheless, for more extensive and more international application of herb medicine, much more effort was needed to precisely clarify the therapeutic mechanism, side effect, toxicity, drug metabolism, and many other aspects of these polyphenols as drugs.

Multiple actions and multiple targets of herb medicine have been widely recognized [156]. Though this article focused on antioxidation of polyphenols, their other pharmacologic actions such as anti-inflammation, antiapoptosis, and angiogenesis promotion were also nonnegligible. Actually, in most literatures reported, the therapeutical effects of polyphenols in ischemic stroke, inflammatory factors, and apoptosis-related proteins were observed altering as well. Though, as introduced above, excess ROS was involved in inflammation and apoptosis, some polyphenols could protect the brain from these harmful factors via oxidative stress-independent mechanisms [21]. Without a doubt, some potential therapeutic mechanisms of polyphenols in treating ischemic stroke still were waiting to be discovered. Above all, antioxidation cooperatively functioned with other relevant effects and mitigated symptoms of ischemic stroke.

## Figures and Tables

**Table 1:** Antioxidant activity of flavonoids in ischemic stroke related studies.

Flavonoids	Cell/animal model	Dosages and methods of administration in animal models	Antioxidation-related indexes	Ref
Baicalein	OGD cell model (SH-SY5Y) MCAO rat model (Wistar)	In vitro: 0.1, 0.25, 0.5, 1, 2, 4, and 8 *μ*M for 12 h In vivo: 2.5, 5, and 10 mg/kg, 7 times for 3 days before surgery, intragastric administration, tail vein injection.	Up: NQO1, Nrf2, GSH-Px, SOD, GSH, and CAT Down: ROS, AMPK, MDA, and 8-OhdG	[30]
	OGD cell model (SH-SY5Y) MCAO rat model (SD)	In vitro: 1, 5, 10, 15, and 20 *μ*M for 12 h In vivo: 30 mg/kg; daily for 7 days, intragastric administration.	Up: MMP	[31]
	Iodoacetic acid-induced oxidative injury cell model (HT22) MCAO New Zealand white rabbit model	In vitro: 1, 2, 5, and 10 *μ*M for 20 h In vivo: 100 mg/kg, subcutaneous injection	Up: cell survival	[32]

Baicalin	Primary rat astrocytes and cortical neurons MCAO rat model (SD)	In vitro: 0.1 to 100 *μ*M In vivo: 100 mg/kg, intraperitoneal injection	Down: SDH and ROS	[33]
	OGD cell model (SH-SY5Y) MCAO rat model (SD)	In vitro: 0.1, 1, and 10 *μ*M for 2 h In vivo: 10, 25, and 50 mg/kg, intravenous administration	Down: superoxide and peroxynitrite	[34]
	Transient global ischemia Mongolian gerbil model	50, 100, and 200 mg/kg; daily for 7 days, intraperitoneal injection	Up: SOD, GSH, and GSH-Px Down: MDA	[77]

EC	H_2_O_2_ and tert-butyl hydroperoxide-simulatedmice embryonic cortical neuronal cells MCAO mouse model (C57BL/6)	In vitro: 0.1, 1, 10, and 100 *μ*M for 6 h In vivo: 2.5, 5, 15, and 30 mg/kg, intragastric administration	Up: HO-1 and nuclear Nrf2 Down: cytoplasmic Nrf2	[78]
	OGD cell model (primary mice cortical neurons) MCAO mouse model (C57BL/6)	In vitro: 50 and 100 *μ*M In vivo: 5, 10, and 15 mg/kg, intragastric administration	Up: HO-1, FTL, and BVR	[79]

ECG	OGD cell model (HBMECs)	0.5, 1, 2, and 4 *μ*M	Up: SOD Down: ROS and MDA	[39]

EGCG	MCAO rat model (SD)	20 mg/kg, intraperitoneal injection.	Up: GSH-Px and SOD Down: NO and MDA	[37]
	MCAO mouse model (C57BL/6)	50 mg/kg, intraperitoneal injection.	Up: Nrf2 and SOD1 Down: GRP78, CHOP, and Caspase 12	[35]
	Dimethylarginine-induced HBMECs injury	20, 40, 60, 80, and 100 *μ*M for 24 h	Down: ROS and MDA	[80]
	MCAO rat model (SD)	40 mg/kg; daily for 3 days, intraperitoneal injection.	Up: GSH, Nrf2, HO-1, GCLC, and GCLM Down: ROS	[36]
	MCAO rat model (SD)	10 mg/kg; one time for 1 h before surgery and daily for Day 4 to Day 7 after surgery, intragastric administration	Up: GSH and SOD Down: NO and MDA	[38]
	Glutamate-induced oxidative injury cell model (HT-22)	1, 10, 50, and 100 100 *μ*M for 10 h	Up: HO-1 Down: ROS	[81]

Quercetin	OGD cell model (hippocampal slices and neuron/glia cultures) MCAO rat model (SD)	In vitro: 10 *μ*M In vivo: 20 mg/kg; daily for 21 days before surgery, intragastric administration	Up: HO-1 Down: MDA	[41]
	MCAO rat model (SD)	10 mg/kg 30 mins before surgery, intraperitoneal injection	Oxidative stress-related proteins	[82]
	MCAO gerbil model	20 mg/kg 30; daily for 21 days before surgery, intragastric administration.	Up: SOD1, SOD2, CAT, and GSH-Px	[83]
	MCAO rat model (SD)	30 mg/kg 30; daily for 14 days, intraperitoneal injection.	Up: GSH, GSH-Px, and GRx Down: lipid peroxidation level	[84]
	MCAO rat model (SD)	30 mg/kg 30 mins before surgery, 0, 24, 48, and 72 h after surgery, intraperitoneal injection	Up: GSH, GR, GSH-Px, GST, SOD, and CAT	[85]

Astragaloside IV	OGD cell model (SH-SY5Y) MCAO rat model (SD)	In vitro: 10, 30, and 60 *μ*g/mL In vivo: 20 mg/kg, intraperitoneal injection	Up: SOD Down: ROS and MDA	[86]
	OGD cell model (neurons)	6.25, 12.5, and 25 *μ*mol/L 3 h before OGD and 24 h after OGD	Up: mitochondrial potential, ATP Down: ROS	[46]
	TIA mouse model (C57BL/6L)	50 mg/kg two times every day for 12 weeks, intragastric administration	Up: T-AOC, SOD, GSH Down: NOX2/4, ROS, and MDA	[45]
	LPS stimulated bEnd.3 cells and C57BL/6 mice	In vitro: 25, 50 and 100 *μ*M In vivo: 12.5, 25, and 50 mg/kg daily for 7 days, intraperitoneal injection	Up: Nrf2, HO-1, and NQO1 Down: ROS	[44]
	OGD cell model (murine cortical neurons)	1, 10, 25, and 50 *μ*M	Up: HO-1, NQO1, and SRXN1 Down: ROS	[87]
	MCAO mouse model (C57/B6)	20 and 40 mg/kg 0, 24, and 48 h after surgery, intraperitoneal injection	Up: GSH-Px and SOD Down: MDA	[43]

Genistein	MCAO rat model (SD)	10 mg/kg daily for 7 days before surgery, intraperitoneal injection	Up: Nrf2 and NQO1 Down: ROS	[47]
	MCAO rat model (SD)	10 mg/kg 5 mins after surgery, intraperitoneal injection	Up: SOD and Nrf1 Down: MDA	[88]
	H_2_O_2_-stimulated primary neurons	0.01, 0.1, and 1 mM	Down: ROS	[48]
	MCAO mouse model (C57/BL6J)	2.5, 5, and 10 mg/kg daily for 14 days before surgery, intragastric administration	Up: SOD and GSH-Px Down: MDA and ROS	[49]

Rutin	Retinoic acid-induced IMR32 cell differentiation	0.1 *μ*M, 10 *μ*M, and 100 *μ*M	Down: ROS	[51]

Hesperidin	MCAO mouse model (C57BL/6J)	100 mg/kg daily for 10 days, intraperitoneal injection	Up: GSH, CAT, SOD, and GSH-Px Down: TBARS	[52]
	MCAO rat model (Wistar)	50 mg/kg daily for 15 days before surgery, intragastric administration.	Up: GSH, CAT, SOD, GR, and GSH-Px Down: TBARS	[53]

Neohesperidin	MCAO rat model (SD)	40 mg/kg daily for 21 days before surgery, intraperitoneal injection.	Up: T-AOC, GSH, SOD, CAT, GSH-Px, GR, POD, Nrf2, and HO-1 Down: MDA, ROS, and MPO	[54]

Apigenin	OGD cell model (PC12)	1, 10, and 20 *μ*M for 6 h	Up: Nrf2, SOD, GSH-Px, CAT, MMP Down: ROS	[59]
	CoCl_2_-induced PC12 MCAO rat model	In vitro: 1-200 *μ*g/mL for 1 h In vivo: 25 mg/kg daily for 7 days, intraperitoneal injection	Up: MMP Down: ROS	[60]

Isoquercetin	OGD cell model (primary hippocampal neurons) MCAO rat model (SD)	In vitro: 20, 40, and 80 *μ*g/mL In vivo: 5, 10, and 20 mg/kg daily for 3 days, intragastric administration	Up: SOD Down: MDA	[62]
	OGD cell model (primary hippocampal neurons) MCAO rat model (SD)	In vitro: 25, 50, and 100 *μ*g/mL In vivo: 50 mg/kg daily for 7 days, intravenous administration	Up: SOD and Nrf2 Down: ROS	[63]
	MCAO rat model (SD)	5, 10, and 20 mg/kg daily for 3 days, intragastric administration	Up: SOD and CAT Down: ROS and MDA	[89]

Isorhamnetin	MCAO mouse model (ICR)	0.5 and 5 mg/kg, 0 and 24 hours after reperfusion, intraperitoneal injection	Up: Nrf2 and HO-1 Down: ROS and MDA	[90]
	Methylglyoxal plus OGD cell model (HBMECs)	10 to 100 *μ*M	Up: GSH Down: ROS	[64]

Phloretin	MCAO rat model (SD)	20, 40, and 80 mg/kg daily for 14 days before surgery, intraperitoneal injection	Up: Nrf2, SOD, GSH, and GSH-Px Down: MDA	[65]

Biochanin A	MCAO rat model (SD)	10, 20, and 40 mg/kg daily for 14 days before surgery, intraperitoneal injection	Up: SOD, GSH-Px, Nrf2, and HO-1 Down: MDA	[66]

Tangeretin	OGD cell model (HBMECs)	2.5, 5, 10, and 20 *μ*M	Up: SOD Down: ROS, MDA, iNOS, and NO	[55]

Morin	MCAO rat model (Wistar)	30 mg/kg, intraperitoneal injection	Down: ROS and MDA	[67]

Breviscapine	MCAO rat model (SD)	20, 50, and 100 mg/kg daily for 7 days before surgery, intraperitoneal injection	Up: SOD, GSH, and CAT Down: MDA	[68]

Hispidulin	MCAO rat model (Wistar)	50 mg/kg daily for 7 days, intraperitoneal injection	Up: Nrf2, SOD, GSH-Px, and CAT Down: MDA, ROS	[69]

Myricetin	MCAO rat model (SD)	1, 5, and 25 mg/kg daily for 7 days before surgery, intragastric administration	Up: SOD and GSH Down: MDA	[91]
	OGD cell model (SH-SY5Y) MCAO rat model (SD)	In vitro: 0.1, 0.33, 1, 3.3 and 10 nM In vivo: 5, 10, and 20 mg/kg 2 h before surgery and 24 h, 48 h after surgery, intragastric administration	Up: Nrf2, HO-1, SOD, CAT, mitochondrial ATP, and MMP Down: ROS, MDA, mitochondrial ROS, and mitochondrial MDA	[70]

Xanthoangelol	MCAO rat model (SD)	50 and 100 mg/kg daily for 3 days, intraperitoneal injection	Up: Nrf2, CAT, SOD and GSH-Px Down: MDA	[71]

Kaempferol	MCAO rat model (SD)	1.75, 3.49, and 6.99 mM daily for 7 days before surgery, intragastric administration	Up: Nrf2, SOD and GSH Down: MDA	[72]

Naringenin	OGD cell model (cortical neurons) MCAO rat model (SD)	In vitro: 20, 40 and 80 *μ*M In vivo: 80 *μ*M, intraperitoneal injection	Up: SOD and Nrf2 Down: MDA and ROS	[57]

Chrysin	MCAO mouse model (C57/BL6)	30 mg/kg, intraperitoneal injection	Up: SOD Down: MDA	[92]
	MCAO rat model (Wistar)	10, 30, and 100 mg/kg daily for 21 days before surgery, intragastric administration	Up: GSH-Px Down: MDA, NO	[93]

Icariin	Angiotensin II- (Ang II-) induced hypertension rat model	10 mg/kg daily for 28 days, intragastric administration	Down: ROS	[73]

Icariside II	OGD cell model (PC12)	12.5, 25, and 50 *μ*M	Up: MMP, Nrf2, NQO1, and HO-1 Down: ROS and Keap1	[74]

Nobiletin	MCAO rat model (SD)	10 and 25 mg/kg daily for 3 days before surgery, intraperitoneal injection	Up: Nrf2, HO-1, GSH, and SOD1 Down: MDA	[58]

Xanthohumol	OGD cell model (primary neurons) MCAO rat model (SD)	0.5 *μ*g/mL 0.4 mg/kg 10 mins before surgery, intraperitoneal injection	Up: CAT, SOD, and Nrf2 Down: ROS, MDA, 4-HNE, 8-OhdG, and GSSSG/GSH	[94]

## Data Availability

No data were used to support this review article.
